# The relationship between personality and the response to acute psychological stress

**DOI:** 10.1038/s41598-017-17053-2

**Published:** 2017-12-04

**Authors:** Yuanyuan Xin, Jianhui Wu, Zhuxi Yao, Qing Guan, André Aleman, Yuejia Luo

**Affiliations:** 1 Shenzhen Key Laboratory of Affective and Social Cognitive Science, Shenzhen University, Shenzhen, 518060 China; 2 Institute of Psychology, Chinese Academy of Sciences, Beijing, 100101 China; 3 University of Chinese Academy of Sciences, Beijing, 100101 China; 4 University Medical Center Groningen, University of Groningen, Groningen, 9713GZ The Netherlands

## Abstract

The present study examined the relationship between personality traits and the response to acute psychological stress induced by a standardized laboratory stress induction procedure (the Trier Social Stress Test, TSST). The stress response was measured with a combination of cardiovascular reactivity, hypothalamic–pituitary–adrenal axis reactivity, and subjective affect (including positive affect, negative affect and subjective controllability) in healthy individuals. The Generalized Estimating Equations (GEE) approach was applied to account for the relationship between personality traits and stress responses. Results suggested that higher neuroticism predicted lower heart rate stress reactivity, lower cortisol stress response, more decline of positive affect and lower subjective controllability. Individuals higher in extraversion showed smaller cortisol activation to stress and less increase of negative affect. In addition, higher openness score was associated with lower cortisol stress response. These findings elucidate that neuroticism, extraversion and openness are important variables associated with the stress response and different dimensions of personality trait are associated with different aspects of the stress response.

## Introduction

Human beings inevitably experience stressful events in their daily life. An acute stressor can trigger the body response in a variety of ways, including a rapid cardiovascular activation (e.g., heart rate (HR) increase, blood pressure increase) dominated by the sympathetic-adrenal-medullary (SAM) axis^1^ and a relatively slow increase of corticosteroid levels exerted by the hypothalamic-pituitary-adrenal (HPA) axis^2^. Acute stress also triggers subjective experiences, for example, perceived stress, positive and negative affect and sense of uncontrollability^3,4^.

Every coin has two sides and acute stress also has its “dual nature”. On the one hand, acute stress has a protective and adaptive function, facilitating rapid reallocation of resources and improving survival chances in a challenging environment. On the other hand, acute stress also suppresses cognitive functions such as executive function and may in the long-term negatively affect the risk of developing psychiatric and physiological problems, including depression, anxiety, schizophrenia, various addictive behaviours and cardiovascular diseases^5^. Moreover, excessive acute stress may lead to post-traumatic stress disorder^6^ and magnify long-lasting changes in cholinergic gene expression^7^.

Notably, there are considerable individual differences in stress responses with some people showing maladaptive responses, whereas others are more resilient to the same stressor. Recently, studies have increasingly focused on individual difference in stress response. For example, studies suggest that the stress response may vary according to sex^8–10^, genetically predisposition^11^, personality^12^, and mindset^13^. Arguably, how to predict individual variability of stress responses, and resilience and vulnerability to stress-related negative effects, could be viewed as the most important topic in this realm (for a review, see^5^).

Specifically, emerging evidence suggests personality traits have been found to be significant predictive factors in individual differences in stress responses. It is demonstrated that neuroticism is associated with attenuated physiological stress responses including cortisol response^3,8,9,14^ and HR^3,15^, and more negative psychological responses such as higher perceived stress, higher negative emotion and lower positive emotionality^3,4^. However, there are also studies found null-results of the relationship between neuroticism and acute stress responses^8,16,17^. A review of the literature on psychological and biological basis of neuroticism also points to inconsistent findings^18^. Some studies also suggest that the other four traits of big five personalities may be associations with acute stress responses. For extraversion, although few studies do not found the relationship between extraversion and acute stress reactivity^3,19,20^, other studies report that higher extraversion is associated with more resilient psychological response to stress, such as more positive subjective feeling and a higher sense of control^4^; a few and inconsistent findings exist in predictive value of extraversion on physiological stress response, with two studies showing opposite results of the relationship between extraversion and cortisol stress response^9,12^ and one showing that higher extraversion predicted blunted HR stress response^21^. For openness, previous results suggest that higher openness is associated with attenuated negative psychological responses to stress, such as lower perceived stress and a smaller increase in negative effect^4,22^. Regarding the physiological response, however, there are inconsistent results in the literature. Some studies report that higher openness is associated with lower cardiovascular reactivity^19,23^. Other studies, however, suggest that higher openness is associated with higher cardiovascular stress responses^3^ and increased cortisol stress response^3,9^. Respect to agreeableness, although Bibbey *et al*.^3^ suggests that participants who are less agreeable had smaller cardiovascular and cortisol stress responses, it is not replicated in other studies^9,12,16^. For conscientiousness, one study find that conscientiousness has positive predictive value in cortisol stress response^16^, while the others do not find the significant relationship^3,9,12^.

Several factors might contribute to the inconsistencies in these findings. First, subjects differed in demographical variables, e.g., sex, age and level of education in different studies, which possibly influenced the individual’s stress responsiveness^8,10,24,25^. Second, stressors were different across these studies, which possibly induced different aspects or levels of stress responses. Some studies induced acute stress with a single public speech^4^, a mental arithmetic^22^ or an interview^19,23^ and some used combined tasks including two cognitive tasks and a public speech^3^. Although stress responses were provoked successfully, issues pertaining to power and validity in the relationship between personality and stress responses warranted more systematic investigations. The Trier Social Stress Test (TSST) is the most commonly used laboratory stress induction procedure in humans and has been proven a useful tool in studies on the relationship between stress and brain function^8^. However, only two studies^9,12^ on the relationship between personality and stress responses utilized TSST as stressor although one did not measure subjective responses to stress^9^ and only middle-aged men included in the other one^12^. Third, different studies used different measurement, which represented distinct aspects of the stress response^26^. Acute stressors can trigger a range of responses, including cardiovascular response, HPA activity and subjective feelings about stress. However, most of previous studies measured only part of them. Bibbey *et al*.^3^ examined all three aspects of stress responses, i.e., cardiovascular activity, cortisol and self-reported impact of stress task, although it used common cognitive tasks (a Stroop task and a mirror tracing task) and a public speech to elicit stress. The stress responses triggered by this combined task was weaker than those reported in studies using TSST or adapted TSST as the acute stressor^3,8,9^. Fourth, inconsistencies might come from problematic scientific bias, e.g., the publication bias^27^ and the internal group dynamics bias^28^, which could possibly hide the truth.

The goal of this study was to examine how personality traits predicted acute stress responses induced by TSST and assessed with multiple measures (SAM-axis, HPA-axis and subjective experiences) in healthy individuals. Based on previous literatures, we predicted that individual differences in personality traits would be predictive of stress responses. Specifically, we hypothesized that higher neuroticism would be related to blunted physiological stress responses (i.e., attenuated HR response and attenuated cortisol response) but more intense affective responses (i.e., larger negative affect (NA) increase, larger positive affect (PA) decrease and lower controllability); higher extraversion would evoke resilient psychological response; higher openness would predict attenuated psychological responses; and we made preliminary hypothesis that agreeableness and conscientiousness would not be associated with stress responses.

## Results

### Personality

Descriptive statistics of the five personality traits are presented in Table 1, including the means and standard deviations (SD). The correlation matrix between these five personality traits by Pearson correlation analysis is also presented in Table 1. No significant relationship between every two personality traits is found.

**Table 1:** Correlation matrix of the Big Five with Pearson correlation analysis (N = 54).

	E	O	A	C	Mean (SD)
N	−0.26	−0.046	−0.12	−0.198	5.83 (2.47)
E		−0.262	0.132	−0.03	7.17 (2.63)
O			0.004	0.163	7.90 (2.30)
A				0.207	11.59 (1.89)
C					11.24 (2.43)

N: neuroticism; E: extraversion; O: openness; A: agreeableness; C: conscientiousness; SD: standard deviation.

### Physiological and psychological stress responses to TSST

**Figure 1:**
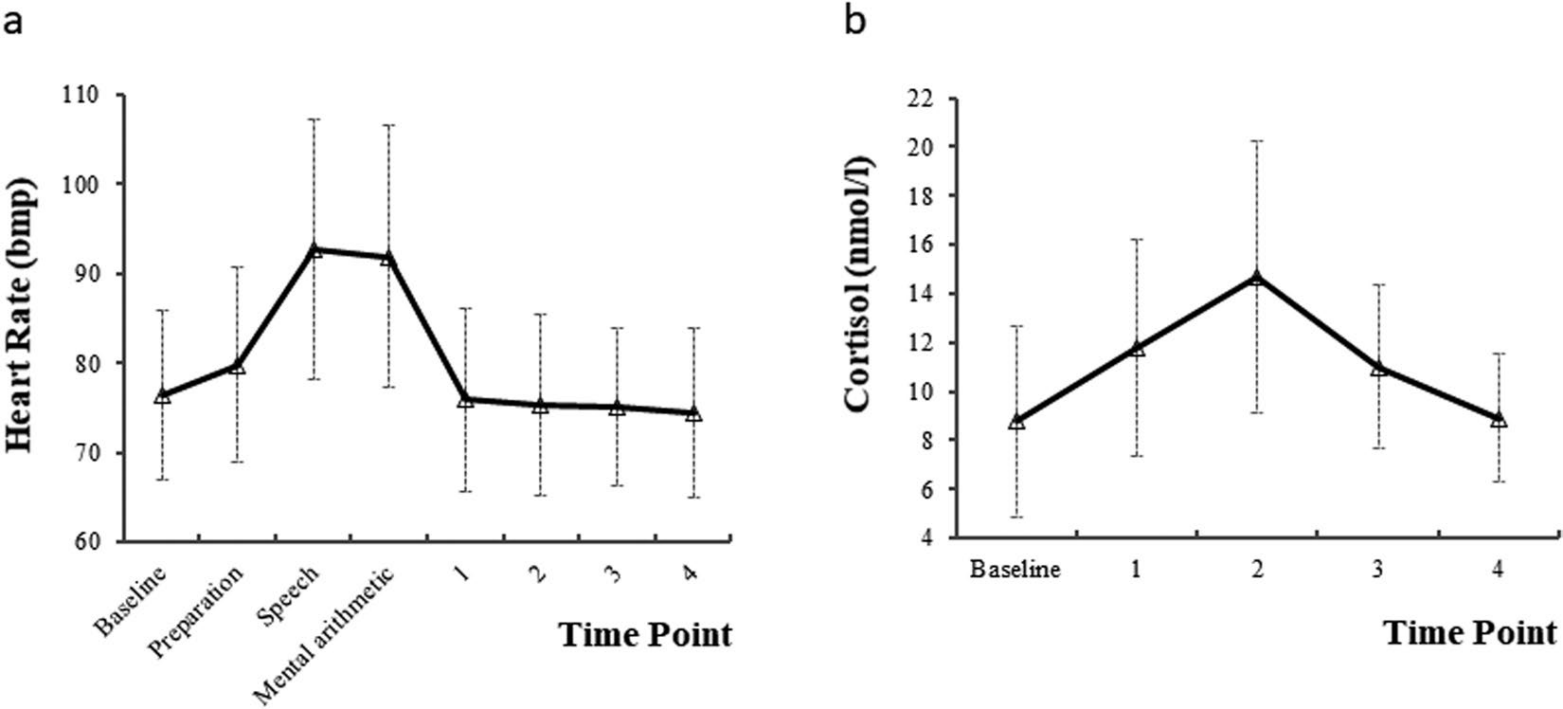
The development of stress responses over time. (**a**) Heart rate curve before, during and after TSST (Trier Social Stress Test). (**b**) Cortisol response before and after the TSST. Error bars shown are standard deviation of the mean.1/2/3/4: at 1 minute, 35 min, 60 min, and 75 min after the onset of the TSST task.

Table 2 presents all repeated observations of HR, Cortisol, PA and NA before, during and after TSST and FoC of TSST (for HR and Cortisol data, also see Fig. 1a and b, respectively).

**Table 2:** Mean (SD) of Heart Rate (HR), Cortisol, Negative Affect (NA), Positive Affect (PA) and Feeling of Control (FoC) measured across time points (N = 54).

Stress Responses	Time Point							
	Baseline	TSST			1	2	3	4
		Preparation	Speech	Mental arithmetic				
HR (bmp)	76.41 (9.52)	79.78 (10.87)	92.69 (14.57)	91.94 (14.61)	75.85 (10.16)	75.31 (10.14)	75.13 (8.77)	74.43 (9.49)
Cortisol (nmol/L)	8.77 (3.92)				11.81 (4.43)	14.69 (5.55)	11.01 (3.37)	8.92 (2.58)
PA	27.56 (6.13)				26.70 (8.13)			
NA	13.44 (4.38)				16.72 (5.61)			
FoC			5.30 (1.93)	5.30 (1.80)				

SD: standard deviation; Time Point 1/2/3/4: at 1 min, 35 min, 60 min, and 75 min after the onset of the TSST task, respectively; HR: heart rate; PA: positive affect; NA: negative affect; FoC: feeling of control.

Compared to Baseline, there was an increase in HR at the period of Preparation (β = 3.37, p < 0.001). Then HR continued increasing, the highest point was reached at the period of Speech (β = 16.3, p < 0.001). Then HR began to decrease at the period of Mental arithmetic but remained higher than Baseline (β = 15.5, p < 0.001). At the Time point 1, 2 and 3 after TSST, HR values were not different from Baseline (all p-values > 0.05) and lower than Baseline at Time point 4 (β = −1.98, p < 0.05).

Cortisol increased at Time point 1 as compared with the Baseline (β = 3.04, p < 0.001) and reached the peak at Time point 2 (β = 5.92, p < 0.001). At Time point 3, cortisol began to decrease, although it was higher than Baseline (β = 2.24, p < 0.001). Then cortisol returned to the level which was similar to Baseline (β = 0.15, p = 0.78) at Time point 4.

There was no significant difference between PA at Baseline and at Time point 1 (β = −0.85, p = 0.22). Compared to Baseline, NA increased significantly at Time point1 (β = 3.28, p < 0.001). There was no significant difference between FoC of Speech and Mental arithmetic (β = −0.007, p = 0.97).

### The relationship between personality traits and stress responses

#### Neuroticism and acute stress responses

The GEE analysis revealed that neuroticism was the only personality trait significant related to HR stress response negatively (β = −0.684, p = 0.008; Odds Ratio (OR) = 0.505, test power = 0.880); neuroticism was also negatively related to Cortisol stress response significantly (β = −0.415, p = 0.001; OR = 0.661, test power = 0.769). In addition, higher neuroticism predicted larger PA decrease (β = −0.36, p = 0.001; OR = 0.698, test power = 0.740) and lower FoC (β = −0.21, p = 0.042; OR = 0.813, test power = 0.56) (for details, see Table 3). With GEE, the univariate regression of neuroticism on stress responses revealed similar results (for details, see Table 4).

**Table 3:** Results of GEE to determine the relationship between acute stress responses and five personality traits (N = 54).

Predictors	Acute stress responses				
	HR	Cortisol	PA	NA	FoC
	β	β	β	β	β
N	−0.68**	−0.42**	−0.36**	0.14	−0.206*
E	−0.16	−0.29*	0.12	−0.20*	0.106
O	−0.43	−0.27*	0.07	−0.25	0.064
A	−0.01	0.29	−0.18	−0.18	−0.117
C	−0.21	−0.18	−0.20	0.21	0.112

HR: heart rate; PA: positive affect; NA: negative affect; FoC: feeling of control; β: the regression coefficient; N: neuroticism; E: extraversion; O: openness; A: agreeableness; C: conscientiousness. Each model also included terms for sex, age, years of education and baseline size. *p < 0.05; **p < 0.01.

**Table 4:** Results of the univariate regressions for five personality traits with GEE (N = 54).

Predictor	Acute stress responses				
	HR	Cortisol	PA	NA	FoC
	β	β	β	β	β
N	−0.596*	−0.338**	−0.344**	0.176	−0.245**
E	−0.047	−0.228*	0.211	−0.31*	0.154
O	−0.497	−0.384**	0.045	−0.29	0.109
A	−0.037	0.237	−0.22	−0.202	−0.027
C	−0.131	−0.090	−0.157	0.15	0.112

HR: heart rate; PA: positive affect; NA: negative affect; FoC: feeling of control; β: the regression coefficient; N: neuroticism; E: extraversion; O: openness; A: agreeableness; C: conscientiousness. Each model also included terms for sex, age, years of education and baseline size. *p < 0.05; **p < 0.01.

#### Extraversion and acute stress responses

The GEE analysis revealed that extraversion was able to positively predict Cortisol stress response (β = −0.292, p = 0.049; OR = 0.746, test power = 0.702). In addition, a negative relationship between extraversion and negative affect was observed (β = −0.20, p = 0.049; OR = 0.819, test power = 0.640) (for details, see Table 3). With GEE, the univariate regression of extraversion on stress responses revealed similar results (for details, see Table 4).

#### Openness and acute stress responses

The GEE analysis showed that openness was a significant personality trait correlated to Cortisol stress response negatively (β = −0.272, p = 0.049; OR = 0.762, test power = 0.688) (for details, see Table 3). With GEE, the univariate regression of openness on stress responses revealed similar results (for details, see Table 4).

#### Agreeableness and acute stress responses

The GEE analysis revealed that there was no significant result of the relationship between agreeableness and acute stress responses (all p-values > 0.05) (for details, see Table 3). With GEE, the univariate regression of agreeableness on stress responses revealed similar results (for details, see Table 4).

#### Conscientiousness and acute stress responses

The GEE analysis revealed that conscientiousness could not predict acute stress responses significantly (all p-values > 0.05) (for details, see Table 3). With GEE, the univariate regression of conscientiousness on stress responses revealed similar results (for details, see Table 4).

## Discussion

The present study investigated how personality traits predicted acute stress responses in healthy individuals using both physiological and psychological measurements. The results indicated significant increases in HR, cortisol and NA induced by TSST, confirming the effectiveness of the TSST in eliciting acute stress responses. Most importantly, with competitive GEE analysis including all the five personality traits, results revealed that individuals’ responses to acute stress differed according to their personality traits, specifically neuroticism, extraversion and openness. Notably, these relationships were achieved after we putted sex, age and years of education into the model.

We found that participants scoring higher in neuroticism showed diminished HR response, attenuated cortisol response, lower PA and less controllability, which were consistent with previous studies^3,4,19,20,22^. These findings imply that more neurotic people have lower physiological responses in both SAM-axis and HPA-axis to acute stress. As neuroticism is a trait originally defined to include anxiety, affective instability, worry, tension and self-pity^27^, it is easy to understand that higher neuroticism scores predicted more intense subjective stress responses, i.e., larger positive effect decrease towards stress and lower feeling of control on stress tasks. Individual with higher neuroticism may experience a higher level of chronic stress, which in turn lead to a down regulation in both the autonomic nervous system^3,28,29^ and HPA system^30,31^.

We found that extraversion was negatively associated with cortisol stress response and NA increment, suggesting that more extraverted individuals had lower HPA-axis stress activity and lower subjective negative response, although we should be cautious about this assumption because these associations became insignificant after Bonferroni correction. These results were consistent with some previous studies^4,12^. Individuals with higher extraversion are more energetic and social, characterized with active emotion coping styles^4,29,32^, more positive affect and less anxiety^27^, which possibly renders them in less negative feeling and smaller cortisol stress reactivity when they encounter a stressor.

We found that greater openness was associated with blunted cortisol stress responding, suggesting that openness predicted decreased HPA axis response to acute stress and played a role in decreasing the slower physiological stress response. This result was different from previous studies showing a positive relationship between openness and cortisol response^3,9^. However, similar findings have been reported for the negative relationship between openness and cardiovascular stress responses in previous literatures^3,19,23^. These inconsistent results regarding the relationship between openness and physiological response to stress suggest that openness may have differential, opposing effects on the stress responses. Individuals with higher openness are characterized as more sensitive, creative and flexible^33,34^.On the one hand, greater levels of sensitivity to their experiences probably lead to greater physiological acute stress responses. On the other hand, a more flexible brain, especially with higher efficiency of functions in the prefrontal cortex in individuals with high openness^35,36^, can negatively regulate HPA response to acute stress^37^. The final response to an acute stressor may depend on the balance between these two opposite effects of openness. We should also be cautious about this result because the association became insignificant after Bonferroni correction. However, we did not find the significant relationship between openness and attenuated negative psychological response, which might require further research.

We did not find significant association between either agreeableness or conscientiousness and stress responses, which was consistent with most other studies^9,12^, although few studies showed the relationship between the two personality factors and physiological stress responses^3,16^. These results suggest that the relationship between the personality trait of agreeableness or conscientiousness and acute stress responses may be less reliable.

The present study had some limitations. First, it should be noted that participants were primarily concentrated on graduates and undergraduate students. It is essential to examine the relationship between personality and acute stress reactivity in samples with a broader range of demographic variables such as age and level of education. Second, although the self-report measure of neuroticism showed significant correlation with both physical and psychological stress responses, an informant-report of neuroticism may provide an even more valid measure to detect the true association. Third, although the present study showed associations between personality traits and acute stress reactivity, there might be other variables explaining and moderating these associations. Future research would possibly examine factors such as coping styles, life events and prefrontal function to elucidate more precise relationships between personality traits and stress responses. Fourth, although the sample size of the present study was sufficient to test for associations between personality traits and stress reactivity, some of the results could not survive the correction of multiple comparisons based on Bonferroni correction, raising the concern for Type I error. It is expected to have a larger sample size for further replication of these findings.

In conclusion, the present study showed that different dimensions of personality predict different aspects of stress responses in the HPA axis, SAM axis and subjective experiences. The results suggest that the personality traits of neuroticism, extraversion and openness had predictive values on acute stress response. These findings pointed to the role of personality traits in individual differences in acute stress response, which may provide insights on understanding how a personality trait characterizes with physiological and psychological stress responses.

## Methods

### Participants

Fifty-four university students (35males, 19 females) aged 18–25years (mean 22.57 ± 1.67) and educated for 13–18 years (mean15.89 ± 1.34) were recruited from universities in Beijing via advertisement. Exclusion criteria included the following: (a) a personal history of psychiatric illnesses, neurological diseases, endocrine disorders or major physiological illness; (b) history of brain damage (e.g., brain surgery, cerebral haemorrhage) or severe head trauma; (c) long-term use of antipsychotic drugs or cortisone; (d) pregnancy; (e) prolonged irregular lifestyle; and (f) major operation in the last 6months. In addition, participants were not in illnesses, taking medicines or suffering from some chronic disease attacks, and they were instructed to avoid staying up during the 3days prior to the study. Female subjects were tested avoiding the ovulation phase of their menstrual cycle. All participants were right handed, non-smokers (no more than five cigarettes a day) or alcoholics (no more than two alcoholic drinks a day) and not with normal or corrected-to-normal vision. This study was approved by the Ethics Committee of Human Experimentation in the Institute of Psychology, Chinese Academy of Sciences. All methods used were in accordance with institutional guidelines and regulations. All participants provided written informed consent and were paid for their participation.

### General procedure

**Figure 2:**
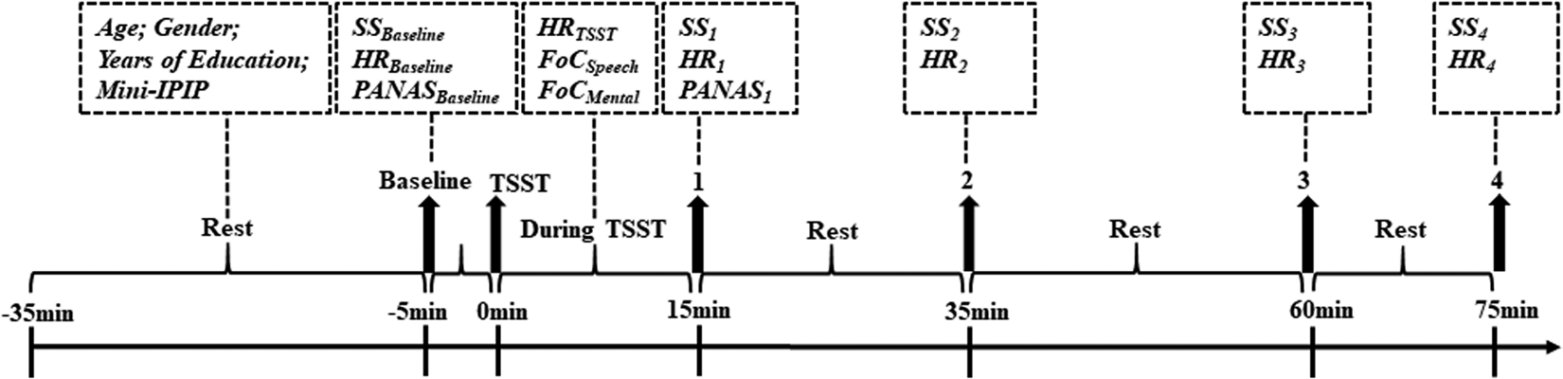
The general procedure of the experiment. The timeline shows the whole data-collecting procedure, including demographic data, saliva sampling (SS), heart rate (HR), the positive and negative affect (PANAS), Feeling of Control (FoC) and stress induction with the Terier Social Stress Test (TSST). Mini-IPIP: mini-International Personality Item Pool.

To control for the circadian rhythm of cortisol levels^38,39^, the experiment was conducted in the afternoon, beginning at approximately 1:30 pm. Participants were instructed to avoid drinking or eating anything except water and abstain from vigorous exercise within two hours before coming to the laboratory in the afternoon. All participants reported that they complied with the requirements. Upon arrival, participants were seated to rest in a quiet room for 30 minutes, during which they completed questionnaires which included demographic variables (age, gender, years of education, etc.) and the Personality Inventory (see details below). This study was part of a large project addressing the psychophysiological variables explaining individual stress responses. After the rest period, participants provided the first salivary sample (SS), heart rate (HR) record and the PANAS for baseline measurements (Time point: Baseline). Then, participants completed the Trier Social Stress Test (TSST, see details below) for stress induction. HR was continuously recorded during the Preparation, Speech, and Mental arithmetic periods of the TSST (Time point: TSST). Immediately after the TSST, the score on feeling of control (FoC) of speech and mental arithmetic were retrospectively collected. One minute after the TSST (Time point: 1), the SS, the HR (continuous recording for 5 minutes), and the PANAS were measured again. Then, participants provided SS, HR records and PANAS at 35 min (Time point: 2), 60 min (Time point: 3), and 75 min (Time point: 4) after the onset of the TSST task. The experimental protocol is illustrated in Fig. 2.

### Questionnaires

#### Personality

Personality was assessed with the Chinese version of the mini-International Personality Item Pool (mini-IPIP)^40^. The mini-IPIP consists of 20 descriptive statements, which subjects rated using a 5-point Likert scale ranging from 1 (disagree strongly) to 5(agree strongly). The twenty items comprise five scales, including extraversion, agreeableness, conscientiousness, neuroticism and openness, each of which has four descriptors. The score of every scale ranges from 5 to 25. The Cronbach’s Alpha is all well above 0.60 and the retest reliability ranges from 0.79 to 0.8^40^.

#### The Positive and Negative Affect Scale (PANAS)

The PANAS^41^ was used to assess participants’ affective state. The scale includes 20 items, with 10 depicting positive affect (interested, excited, inspired and alert) and 10 depicting negative affect (distressed, nervous, scared and upset). The subjects were asked to rate on a 5-point Likert scale labeled very slightly or not at all, a little, moderately, quite a bit, and very much, respectively. The score of either PA or NA ranges from 10 to 50. The PANAS scale has high internal consistency reliability with Cronbach’s Alpha of 0.88 for PA, 0.87 for NA^41^.

#### Feeling of Control (FoC)

Participants assessed their sense of control on the speech task and mental arithmetic task using a visual analog scale, with 0 indicating they felt out of control and 10 indicating a strong sense of control.

### Trier Social Stress Test

An adjusted version of the TSST from Kirschbaum and his co-workers^8^ was used to induce a stressful condition, starting with a 5-min preparation, followed by a 5-min speech and a 5-min mental arithmetic task. The modified TSST was as effective as or even more effective in eliciting cortisol responses than the original TSST^42^. During preparation period, participants were asked to prepare a 5-min speech in which they should defend himself/herself against charges of shoplifting made by store managers. They were allowed to take notes for the speech, but not allowed to speech with the notes. After the preparation, participants completed the speech and mental arithmetic tasks using a microphone and were recorded with a video camera. Three experimenters (two females and one male) with white coats and neutral facial expression were present throughout the TSST. For the mental arithmetic task, the participants were instructed to do a continuous subtraction with a decrement of 13 from 1,022 as quickly and accurately as possible. Once they made an error, they had to restart at 1,022.

### Stress response measurement

Saliva samples were collected using Salivettes (Sarstedt, Rommelsdorf, Germany) and was frozen at −22 °C until analysis. Samples were dissolved and centrifuged at 3,000 rpm for 10 min. Cortisol in saliva was measured with electrochemiluminescence immunoassay (Cobas e 601, Roche Diagnostics, Numbrecht, Germany). The lower sensitivity for cortisol was 0.5 nmol/L. Intra- and inter-assay variations were less than 10%.

HR was recorded by Biopac Amplifier-System (MP150; Biopac, Goleta, CA, USA) with three electrocardiograph electrodes placed on the right side of the neck, and the left and right inner ankles. Signals were recorded at a sample rate of 1,000 Hz. In every time point, HR was calculated by averaging the 5-min continuous recording using the AcqKnowledge software and defined as the number of beats per minutes (bpm).

### Data Analysis

We applied the Generalized Estimating Equations (GEE) approach to account for all the repeated observations of HR, Cortisol and affect stress responses across time and relationships between personality traits^43–45^ and these stress responses. Based on examination of the data by the Quasi-likelihood Independence Criterion (QIC), we assumed an exchangeable working correlation structure for the within-subject variable^46^.

Specifically, we conducted the GEE analyses with HR, Cortisol, PA, NA and FoC as dependent variables to investigate the possible predictor variables, respectively. First, to test if the acute stress was induced successfully, we considered time as a dummy variable to model the development of physiological stress responses over time^45^. Second, to investigate the predictive value of personality on stress responses, five personality traits were included in the model competitively. We also added terms of baseline size, sex, age and years of education in regression models by GEE as they probably influenced the stress responsiveness^8,10,24,25^. At last, univariate regressions for each of the five personality traits were also analysed with GEE.

We used Odds Ratio (OR) to evaluate the effect size of each predictor. The statistical test power analyses were conducted with the GPower software^47^. To avoid the overall Type I error rate, the regression coefficient should be tested at the corrected threshold p < 0.01 based on Bonferroni correction.

All personality and stress response variables were normally distributed as tested by P-P plot. The statistical analyses were accomplished using the statistical package SPSS 20.0 (IBM Corp. Armonk, NY). All reported p-values were two-tailed with the significance level of 0.05.

### Data availability

The datasets generated and analysed during the current study are available from the corresponding author on reasonable request.
